# Analysis of PIK3CA Mutations and Activation Pathways in *Triple Negative* Breast Cancer

**DOI:** 10.1371/journal.pone.0141763

**Published:** 2015-11-05

**Authors:** Paolo Cossu-Rocca, Sandra Orrù, Maria Rosaria Muroni, Francesca Sanges, Giovanni Sotgiu, Sara Ena, Giovanna Pira, Luciano Murgia, Alessandra Manca, Maria Gabriela Uras, Maria Giuseppina Sarobba, Silvana Urru, Maria Rosaria De Miglio

**Affiliations:** 1 Department of Clinical and Experimental Medicine, University of Sassari, Sassari, Italy; 2 Department of Pathology, “A. Businco” Oncologic Hospital, ASL Cagliari, Cagliari, Italy; 3 Epidemiology and Medical Statistics Unit, Department of Biomedical Sciences, University of Sassari, Research, Medical Education and Professional Development Unit, AOU Sassari, Sassari, Italy; 4 Department of Biomedical Sciences, University of Sassari, Sassari, Italy; 5 Department of Pathology, AOU Sassari, Sassari, Italy; 6 Department of Medical Oncology, ASL Nuoro, Nuoro, Italy; 7 Biomedicine Sector, Center for Advanced Studies, Research and Development in Sardinia Technology Park Polaris, Cagliari, Italy; Peter MacCallum Cancer Centre, AUSTRALIA

## Abstract

**Background:**

Triple Negative Breast Cancer (TNBC) accounts for 12–24% of all breast carcinomas, and shows worse prognosis compared to other breast cancer subtypes. Molecular studies demonstrated that TNBCs are a heterogeneous group of tumors with different clinical and pathologic features, prognosis, genetic-molecular alterations and treatment responsivity. The PI3K/AKT is a major pathway involved in the regulation of cell survival and proliferation, and is the most frequently altered pathway in breast cancer, apparently with different biologic impact on specific cancer subtypes. The most common genetic abnormality is represented by PIK3CA gene activating mutations, with an overall frequency of 20–40%. The aims of our study were to investigate PIK3CA gene mutations on a large series of TNBC, to perform a wider analysis on genetic alterations involving PI3K/AKT and BRAF/RAS/MAPK pathways and to correlate the results with clinical-pathologic data.

**Materials and Methods:**

PIK3CA mutation analysis was performed by using cobas® PIK3CA Mutation Test. EGFR, AKT1, BRAF, and KRAS genes were analyzed by sequencing. Immunohistochemistry was carried out to identify PTEN loss and to investigate for PI3K/AKT pathways components.

**Results:**

PIK3CA mutations were detected in 23.7% of TNBC, whereas no mutations were identified in EGFR, AKT1, BRAF, and KRAS genes. Moreover, we observed PTEN loss in 11.3% of tumors. Deregulation of PI3K/AKT pathways was revealed by consistent activation of pAKT and p-p44/42 MAPK in all PIK3CA mutated TNBC.

**Conclusions:**

Our data shows that PIK3CA mutations and PI3K/AKT pathway activation are common events in TNBC. A deeper investigation on specific TNBC genomic abnormalities might be helpful in order to select patients who would benefit from current targeted therapy strategies.

## Introduction

Breast cancer (BC) is the most frequent malignant tumor in females and the commonest cause of cancer death among women worldwide [1]. Recently, our knowledge on BC biology has strongly been improved, with significant increase in personalized treatment options. Gene expression profiling studies have established the heterogeneous nature of BC, which might be considered as a collection of distinct “*intrinsic*” subtypes, based on specific genetic alterations involving different oncogenic pathways [2–8].

Triple Negative Breast Cancer (TNBC), which accounts for 12–24% of all breast carcinomas, is defined by the lack of expression of estrogen, progesterone receptors (ER, PgR) and HER2 [9]. TNBCs are a heterogeneous group of tumors with different clinical-pathologic features, genetic-molecular alterations and treatment responsivity [10]. Specifically, molecular profiling studies demonstrated that a high percentage of TNBCs showed basal-like features, whereas the remainders are biologically and genetically different subtypes [11,12]. Indeed, although TNBCs are prevalently categorized as high grade invasive ductal variants [13], other less common or special subtypes, such as metaplastic, medullary, adenoid cystic tumors are still included among TNBC, from which they substantially differ in terms of biologic behavior and clinical course [11].

The PI3K/AKT is a major pathway involved in the regulation of cell processes, such as survival, growth, motility and metabolism, and it is known to be deregulated in a large variety of human cancers [14]. The PI3K/AKT signaling pathway is the most recurrently altered pathway in BC, apparently with different biologic impact on specific cancer subtypes [14]. In this pathway, the most common genetic abnormality is represented by activating mutations in Phosphatidylinositol-4-5-bisphosphate-3-kinase catalytic subunit-α (PIK3CA) gene, with a reported frequency of 20–40% in BC [15,16]. PIK3CA mutations are more prevalent in ER/PgR positive (35%) and HER2-overexpressing BC (23%) than in TNBC (ranging from 5% to 13.2%) [17–22]. Recently, Kriegsmann et al. demonstrated a high frequency of PI3K pathway alterations, comprising mainly PIK3CA mutations, in a large series of TNBC [23]; moreover, PIK3CA mutations were also identified in TNBC-homologous molecular subtype, i.e. basal-like BC [8].

Due to their Triple Negative nature, chemotherapy is currently the mainstay of systemic treatment for patients with TNBC; nevertheless, the sensitivity of these tumors to chemotherapy is low, and only 30% of patients achieve a complete pathological response with neoadjuvant chemotherapy. Therefore, TNBC subtypes represent a priority target of therapeutic research. [8,24].

The aim of our study was to investigate PIK3CA mutations in a large series of TNBC. Furthermore, we performed a wide analysis on genetic alterations that up-regulate PI3K/AKT and BRAF/RAS/MAPK pathways, to correlate with clinical-pathologic data.

## Materials and Methods

Our experimental study was approved by ASL Sassari Bioethical Committee, which also waived the need for written informed consent from the patients, according to the Italian legislation concerning the guidelines for the performance of retrospective observational studies; however, breast tissue samples were fully anonymized prior of any authors’ access.

Tumor samples were selected from the Histopathology Departments archives of Cagliari and Sassari (Italy). Specifically, we retrieved 97 primary TNBC, consecutively identified in between a pool of 650 primary BC, diagnosed in about 1-year routine activity. Moreover, we selected 36 consecutive non-TNBC, categorized as 15 Luminal-A-like (ER+, PgR+, Her2-), 12 Luminal-B-like (ER+, PgR+/-, Her2+) and 9 Her2-positive tumors (ER-, PgR-, Her2+), respectively. All cases were reviewed by two experienced pathologists, and categorized according to current WHO classification [25].

From representative formalin-fixed, paraffin-embedded (FFPE) specimens, 3μm-thick tissue sections were cut for haematoxylin and eosin stains (H & E) and immunohistochemical analysis. Additional consecutive sections were also obtained for genetic analysis. FFPE specimens from control group (non-TNBC tumors) were utilized only for PIK3CA mutational analysis.

### Real-Time PCR to detect PIK3CA gene mutations

To detect PIK3CA mutations a Real-Time PCR procedure was used. TNBC and BC control group were processed for genomic DNA isolation using cobas® DNA Sample Preparation Kit (Roche Mannheim, Germany) following the manufacturer's instructions. Briefly, deparaffinized 5 μm section, containing at least ≥10% of tumor cells, was used for the extraction process. The amount of genomic DNA mixture was spectrophotometrically determined (NanoDrop2000, Thermo Fisher Scientific, Waltham, MA USA) and adjusted to a fixed concentration to be added to the amplification/detection.

The PCR Real-Time cobas® PIK3CA Mutation Test kit (Roche) uses a pool of primers divided into three different mixes for each samples and controls that define specific base-pair (bp) sequences that range from 85 to 155 bp in PIK3CA exons 1, 4, 7, 9, and 20. An additional primer pair, targeting a conserved 167 bp region in PIK3CA exon 3, provides a full process control. A derivative of Thermus species Z05-AS1 DNA-polymerase is utilized for amplification. Selective amplification of target nucleic acid from the sample is achieved in the cobas® PIK3CA Mutation Test by the use of AmpErase (uracil-N-glycosylase) enzyme and deoxyuridine triphosphate (dUTP).

The target DNA was amplified and detected on the cobas z480 analyzer (Roche) using the RT-PCR-based amplification and detection reagents provided in the cobas® PIK3CA Mutation Test kit (Roche). The validated instrument software of the cobas 4800 system uses a specific and tested algorithm for the interpretation of the results, through the automatic and standardized analysis of the specific kinetic of single curve, in order to guarantee the correct interpretation of the amplified curves. No operator mediated evaluation or interpretation is needed and/or possible.

### Gene sequencing to detect EGFR, AKT1, BRAF and KRAS mutations

Gene mutation analyses were performed on specific exons which are known to harbor the most frequent and significant mutations for each gene: exons 18, 19, 20, and 21 for EGFR, exon 2 for AKT1, exon 15 for BRAF and exons 2 and 3 for KRAS. Selected primers (Eurofins MWG, Synthesis GmbH, Munich, Germany) used for the amplification and sequencing reaction are summarized in Table 1.

**Table 1:** Selected Primers for PCR and gene sequencing.

Primers	Sequence	Annealing temperature	Base pair
BRAF F exon 15	TCATAATGCTTGCTCTGATAGGA	55.5°C	185
BRAF R exon 15	GGCCAAAAATTTAATCAGTGGA		
K-RAS F2 exon 2	GTTTGTATTAAAAGGTACGGTGGA	58°C	270
K-RAS R2 exon 2	ATCAAAGAATGGTCCTGCAC		
K-RAS F2 exon 3	CCAGACTGTGTTTCTCCCTTC	59°C	288
K-RAS R2 exon 3	TATGCATGGCATTAGCAAAGACTC		
PIK3CA F exon 9	TCCAGTCACTGTGCTGCTTC	56.8°C	487
PIK3CA R exon 9	GCAAGGGAAAAGGGAGTCTT		
PIK3CA F1-nested exon 9	TTGCTTTTTCTGTAAATCATCTGTG	55.5°C	270
PIK3CA R2-nested exon 9	GCCAAATTCAGTTATTTTTTCTGT		
PIK3CA F exon 20	TGACATTTGAGCAAAGACCTG	59.4°C	445
PIK3CA R exon 20	GGATTGTGCAATTCCTATGC		
PIK3CA F-hemi exon 20	AGGTTTCAGGAGATGTGTTAC	59.4°C	372
PIK3CA R exon 20	GGATTGTGCAATTCCTATGC		
EGFR F exon 18	GCTTGCAAGGACTCTGGGCT	62°C	360
EGFR R exon 18	CCAAACACTCAGTGAAACAAAGAG		
EGFR F exon 19	GTGCATCGCTGGTAACATCCA	55°C	306
EGFR R exon 19	CATTTAGGATGTGGAGATGAGC		
EGFR F exon 20	GAAACTCAAGATCGCATTCATGC	60°C	379
EGFR R exon 20	GCAAACTCTTGCTATCCCAGGAG		
EGFR F exon 21	CTAACGTTCGCCAGCCATAAGTCC	57°C	370
EGFR R exon 21	GCTCACCCAGAATGTCTGGA		
AKT1 F exon 2	AGGCACATCTGTCCTGGCAC	61°C	263
AKT1 R exon 2	AAATCTGAATCCCGAGAGGCC		

Five 10 μm-thick consecutive sections from TNBC specimens were prepared, and tumors were macro-dissected with a scalpel blade under sterile conditions, using corresponding Haematoxylin & Eosin stained sections as a guide. DNA was extracted using the QIAamp DNA FFPE Tissue Kit (Qiagen, Hilden, Germany) in accordance with the manufacturer’s instructions. To obtain genomic DNA 10μl of RNase A (20mg/ml, Rnase PureLink, Life Technologies, Carlsbad, CA, USA) were applied to the silica membrane to digest contaminating RNA. We assessed the quantity and the quality of nucleic acids spectrophotometrically, as described above. Gene sequencing analysis was executed as previously reported [26].

### Immunohistochemistry

The immunohistochemistry was performed using specific antibodies against mouse monoclonal Androgen Receptor (AR, clone 2F12, dilution 1:25, Novocastra, Dublin, OH, USA) [27], mouse monoclonal Cytokeratin 5/6 (CK5/6, Clone CK5/6.007, dilution 1:100, Biocare Medical, Concord, CA, USA) [28], mouse monoclonal p-AKT (Clone HP18, dilution 1:75, Novocastra), [29], rabbit monoclonal p-p44/42 MAPK (Clone 20G11, dilution 1:100, Cell Signaling Technology, Boston, MA, USA), [30] and mouse monoclonal PTEN (Clone 6M2.1, dilution 1:200, DakoCytomation, Glostrup, Denmark) [31]. Immunoreactions were obtained by incubating sections with specific primary antibodies for 15 minutes. Immunodetection was performed using a non-biotin highly sensitive system (EnVision Peroxidase Detection System, Dako), preventing possible false-positive staining due to endogenous biotin present in the tissue. The slides were then incubated with substrate chromogen solution diaminobenzidine (DAB) for 10 minutes and counterstained with haematoxylin. Specifically, mouse monoclonal EGFR (Clone 2-18C9) immunoreaction was executed using EGFR pharmDx™ Kit (DakoCytomation) [32], according to manufacturer’s instructions.

### Evaluation of Immunohistochemical Staining

AR expression was interpreted as positive if at least 1% immunostained tumor nuclei were detected in the sample, according with ASCO/CAP recommendations for immunohistochemical testing of hormone receptors in BC [33].

EGFR was considered positive when ≥ 1% of neoplastic cells exhibited positivity, according to manufacturer’s instructions; CK5/6 were considered positive when ≥ 5% of neoplastic cells exhibited positivity; moreover, the results were scored semi-quantitatively including intensity (0, negative; 1+, weak; 2+, moderate; 3+, strong).

p-AKT, p-p44/42 MAPK and PTEN staining were scored semiquantitatively based on staining intensity (0–3) and percentage of stained cells (0–100) using the histo-score (H-score; range 0–300) [34]. PTEN loss cut-off corresponding to the H-score ≤10 was used. In the same way, p-AKT and p-p44/42 MAPK with H-score ≤10 were defined as negative. A score ranging from 11 to 100 was considered as weakly positive (score 1), from 101 to 200 moderately positive (score 2), and from 201 to 300 strongly positive (score 3). Finally, subcellular localization of immunostaining was also assessed for each antibody in each positive tumor.

Subcellular localization of immunostaining has also been assessed for each positive tumor case.

### Statistical analysis

Statistical analysis was carried out with STATAR13 (StataCorp, College Station, TX, USA). Shapiro-Wilk normality test was used to assess the parametric distribution of the quantitative variables. Median and inter-quartile range and frequencies were used to summarize quantitativeand qualitative variables, respectively. Statistical differences between individuals with and without PIK3CA mutations for quantitative and qualitative variables were evaluated performing the Mann-Whitney U test and Chi2 or Fisher's Exact Test when appropriated, respectively. Logistic regression analysis, both univariate and multivariate, was carried out to assess the association between mortality after 5 years of follow-up and the epidemiological, clinical, and molecular variables. The statistical significance was set-up at <0.05.

## Results

### Clinic-pathologic features

One hundred and thirty-three primary BC were included in the study, specifically 97 TNBC and 36 non-TNBC. TNBC samples were characterized by ER, PR, and HER2 negativity, with ki67 proliferation index ranging from 6% to 90% of neoplastic cells. Patients’ age ranged from 27 to 92 years (mean: 56), tumor size varied between 8 and 140 mm (mean: 30.5 mm). The clinical-pathologic data of TNBC included in this study are reported in Table 2.

**Table 2:** Clinic-pathologic and biologic data of the TNBC patients according to mutational status of PIK3CA.

Variables	Total cohort	PIK3CA mutations		p-value
		Wildtype	Mutated	
Age, median (IQR)	57 (43–67)	54 (42–64)	66 (55–77)	**0.006**
Tumor size, median (IQR)	25 (15–40)	24 (15–35)	25 (17–45)	0.29
Ductal histologic subtype, n (%)	81 (83.5)	62 (83.8)	19 (82.6)	1.00
Lobular histologic subtype	3 (3.1)	1 (1.4)	2 (8.7)	0.14
Other histologic subtypes	13 (13.4)	11 (14.9)	2 (8.7)	0.73
pT1, n (%)	38 (39.6)	29 (39.7)	9 (39.1)	0.21
pT2	40 (41.7)	33 (45.2)	7 (30.4)	
pT3	13 (13.5)	7 (9.6)	6 (26.1)	
pT4	5 (5.2)	4 (5.5)	1 (4.4)	
pN0-N1, n (%)	70 (76.9)	57 (81.4)	13 (61.9)	0.07
pN2-N3	21(23.1)	13 (18.6)	8 (38.1)	
Stage I, n (%)	24 (24.7)	19 (25.7)	5 (21.7)	0.14
Stage II	43 (44.3)	36 (48.7)	7 (30.4)	
Stage III	30 (30.9)	19 (25.7)	11 (47.8)	
Grade I, n (%)	4 (4.1)	4 (5.4)	0 (0.0)	**0.03**
Grade II	16 (16.5)	8 (10.8)	8 (34.8)	
Grade III	77 (79.4)	62 (83.8)	15 (65.2)	
Ki67, median (IQR)	45 (25–70)	60 (30–70)	25 (12–60)	**0.004**
Mortality (5-years), n (%)	8 (12.1)	4 (8.0)	4 (26.7)	0.08

IQR: interquartile range; n: number

### Mutational Analysis

Mutational analysis of PIK3CA gene was achieved in all TNBC and BC control groups. PIK3CA somatic missense mutations were detected in 23/97 of TNBC (23.7%) and in 12/36 non-TNBC (33.3%), in detail, 6/15 Luminal-A-like (40%), 3/12 Luminal-B-like (25%) and 3/9 HER2-positive tumors (33.3%); among the TNBC, only one tumor harbored mutations in both exons 9 and 20.

As regards the analysis of PIK3CA exon 9, involving the helical domain of PIK3CA gene, our results showed the presence of three hotspot mutations “E542K”, “E545X” and “E546X”, which were revealed in 7/97 TNBC (7.2%). The mutational analysis of PIK3CA exon 9 on non-TNBC demonstrated the presence of “E545X” mutation, involving the 3/36 of tumors (8.3%), with mutations in 2/15 Luminal-A-like and 1/9 HER2-positive tumors, whereas the subtype Luminal-B-like did not show any mutation on exon 9.

The analysis of PIK3CA exon 20, involving the kinase domain of PIK3CA gene, showed the presence of one hotspot mutation “H1047X”, which were identified in 16/97 TNBC (16.5%). The mutational analysis of PIK3CA exon 20 on non-TNBC demonstrated the presence of two hotspot mutations, “H1047X” and “G1049R”, involving 8/36 of tumors (22.3%), with mutations in 4/15 Luminal-A-like, 2/12 Luminal-B-like and 2/9 HER2-positive tumors. In a single Luminal-B-like tumor a mutation on PIK3CA gene exon 4 was also identified (2.7%), “N345K”, located in the C2 domain of PIK3CA, promoting its activity. Finally, exon 20 was confirmed the most frequently mutated in all BC analyzed [35,36].

Genomic DNA sequencing of EGFR exons 18, 19, 20, and 21, AKT1 exon 2, BRAF exon 15 and KRAS exons 2 and 3 failed to demonstrate mutations in any of the TNBC analyzed.

### Immunohistochemical analysis

Immunohistochemistry was performed to define TNBC subtypes and to analyze downstream signaling pathways.

Androgen Receptor (AR) expression was identified in 20/92 TNBC (21.7%).

The immunoreactivity for basal markers, EGFR and CK5/6 was reported as membranous or membranous-cytoplasmic. EGFR expression was appreciable in 69.1% of TNBC, with staining intensity ranging from 1+ to 3+, and the percentages of positive cells varying from 5% to 90%. No immunoreactivity was observed in non-neoplastic tissues. CK5/6 expression was appreciable in 63.9% of TNBC, with staining intensity ranging from 1+ to 3+, and the percentages of positive cells varying from 5% to 95%. Twenty-five TNBC were negative for both basal markers and were considered as TNBC without basal-like features.

The analysis of downstream signaling pathways, including p-AKT and p-p44/42 MAPK, showed nuclear staining and nuclear and/or cytoplasmic staining, respectively. p-AKT expression was absent in 22.8% of TNBC (score 0), whereas 32.7% presented weak (score 1), 21.7% moderate (score 2) and 22.8% strong expression of p-AKT. p-p44/42 MAPK expression was absent in 42.3% of TNBC (score 0), whereas 26.8% presented weak (score 1), 18.6% moderate (score 2) and 12.3% strong (score 3) expression of p-p44/42 MAPK. PIK3CA mutations were prevalently associated with strong expression of pAKT, namely 12/21 (57.1%) TNBC with mutated PIK3CA showed score 3, 4/20 (20%) TNBC were score 2, 7/30 (23.3%) TNBC were score 1, whereas no TNBC (0/21) with mutated PIK3CA was score 0.

**Fig 1:**
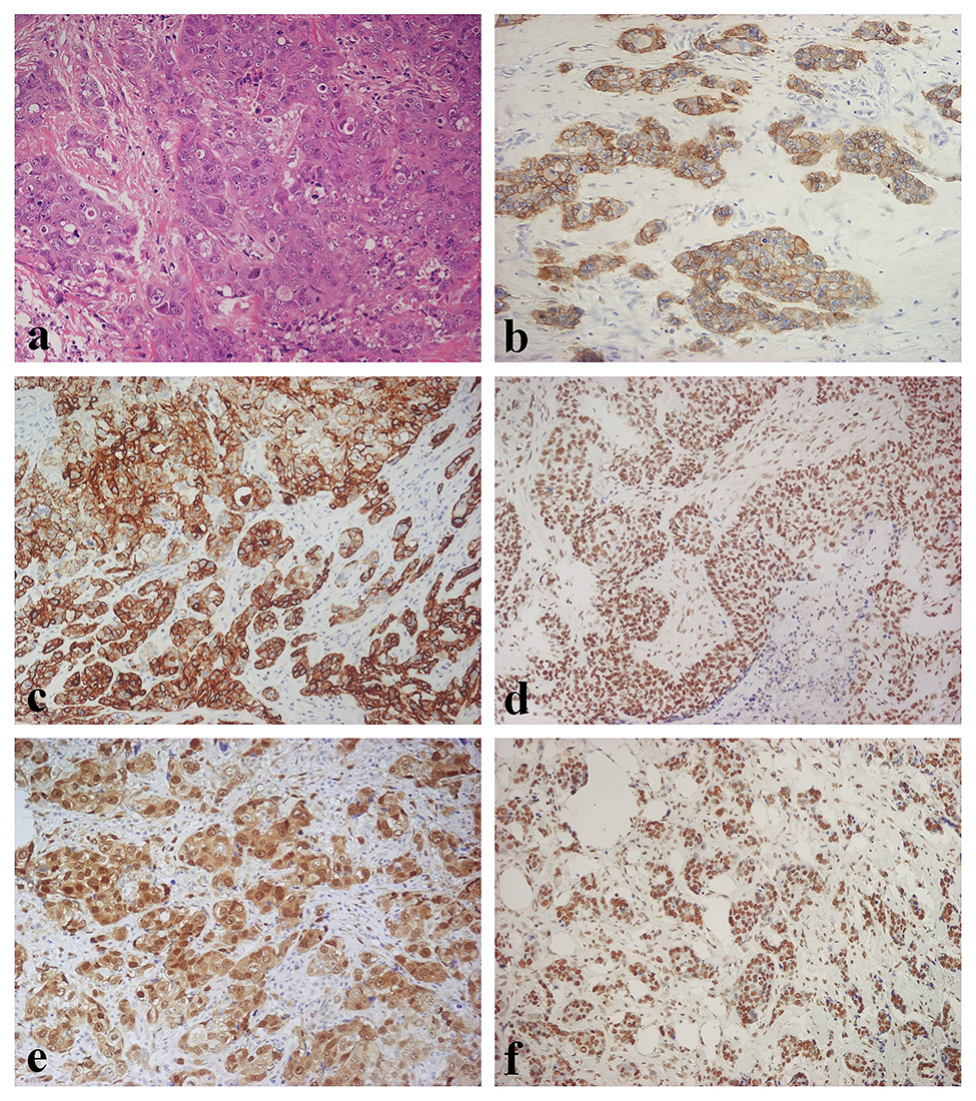
Morphologic and immunohistochemical features of Triple Negative Breast Cancer. (A) Haematoxylin & Eosin stain illustrates a Triple Negative variant with features of high grade invasive ductal carcinoma (original magnification 100X); (B) Immunohistochemistry for EGFR displaying diffuse and moderate membranous and membranous-cytoplasmic immunoreactivity (original magnification 100X); (C) Immunohistochemistry for CK5/6 showing diffuse and intense cytoplasmic immunoreactivity (original magnification 100X); (D) Immunohistochemistry for p- AKT showing diffuse and intense nuclear immunoreactivity (original magnification 100X); (E) Immunohistochemistry for p-p44/42 MAPK displaying diffuse and intense nuclear-cytoplasmic immunoreactivity (original magnification 100X); (F) Immunostaining for PTEN showing diffuse and intense nuclear immunoreactivity (original magnification 100X).

PTEN showed nuclear staining. PTEN loss was identified in 11.3% of TNBC (score 0), all with basal-like features; whereas 21.6% presented weak (score 1), 39.3% moderate (score 2) and 27.8% strong expression (Table 3, Fig 1).

**Table 3:** Immunostaining data of the TNBC patients according to mutational status of PIK3CA.

Variables		Total cohort	PIK3CA mutations		p-value
Antibody	Staining		Wildtype	Mutated	
AR, n (%)	Positive	20 (21.7)	10 (14.5)	10 (45.5)	**0.002**
EGFR, n (%)*	0	30 (30.9)	22 (29.7)	8 (34.8)	0.59
	1+	18 (18.6)	16 (21.6)	2 (8.7)	
	2+	26 (26.8)	19 (25.7)	7 (30.4)	
	3+	23 (23.7)	17 (23.0)	6 (26.1)	
pAKT, n (%)§	0	21 (22.8)	21 (30.4)	0 (0.0)	**<0.0001**
	1	30 (32.7)	23 (33.4)	7 (30.4)	
	2	20 (21.7)	16 (23.2)	4 (17.4)	
	3	21 (22.8)	9 (13.0)	12 (52.2)	
p-p44/42MAPK, n (%)§	0	41 (42.3)	33 (44.6)	8 (34.8)	0.31
	1	26 (26.7)	18 (24.3)	8 (34.8)	
	2	18 (18.6)	12 (16.2)	6 (26.0)	
	3	12 (12.4)	11 (14.9)	1 (4.4)	
pTEN, n (%)§	0	11 (11.3)	9 (12.2)	2 (8.7)	0.08
	1	21 (21.7)	19 (25.7)	2 (8.7)	
	2	38 (39.2)	30 (40.5)	8 (34.8)	
	3	27 (27.8)	16 (21.6)	11 (47.8)	

n: number

*: expressed as immunohistochemical intensity

§: expressed as H-score

Tumors with PTEN loss (score 0) showed consistent but variable pAKT expression in 11/11 TNBC, whereas tumors with PTEN scores 1 to 3 showed variable pAKT expression in 61/81 TNBC.

### Mutational profiling and association analysis

No significant differences were detected between TNBC and non-TNBC PIK3CA mutational status (23.7% *vs* 33.3%, p = 0.262).

The statistical differences between PIK3CA mutational status and standard clinical, pathological and biological features of TNBC were analyzed (Table 2). Briefly, PIK3CA mutations were significantly higher in older individuals (mean: 66 years *vs* 54 years; p = 0.006). However, no significant differences were obtained comparing other variables, such as tumor size, histologic type (although ductal carcinomas were prevalently represented) pT, pN, Stage, and OS. Interestingly, TNBC showing PIK3CA mutations were prevalently of lower grade (p = 0.03) with lower proliferation index (p = 0.004).

A statistically significant association was obtained between PIK3CA mutational status, AR expression (p = 0.002) and p-AKT expression (p = 0.0001). No statistical differences were observed between PIK3CA mutational status and immunohistochemical expression of EGFR, p-p44/42 MAPK, and PTEN, as summarized in Table 3.

A logistic regression analysis was carried out in order to assess the impact of established clinic-pathologic prognostic predictors on TNBC overall patient survival. Although lower Ki67 values were significantly associated with a better overall patient survival (p = 0.015), a multivariate model analysis did not confirm Ki67 value as an independent prognostic factor (Table 4).

**Table 4:** Association between overall survival and clinic-pathological and molecular variables.

Variables	Univariate analysis	p-value	Multivariate analysis	p-value
	OR (95% CI)		OR (95% CI)	
Age	1.05 (0.99–1.08)	0.09	-	-
Tumor size	1.02 (0.99–1.05)	0.12	-	-
Ductal histologic subtype	0.55 (0.10–3.17)	0.51	-	-
Lobular histologic subtype	8.14 (0.46–145.18)	0.15	-	-
Other histologic subtypes	0.89 (0.10–8.26)	0.92	-	-
pT	1.45 (0.62–3.41)	0.39	-	-
pN	1.94 (0.41–9.2)	0.41	-	-
Stage	1.41 (0.50–3.99)	0.52	-	-
Grade	1.09 (0.28–4.22)	0.90	-	-
Ki67	0.94 (0.88–0.99)	**0.015**	0.95 (0.86–1.05)	0.33
AR	4.09 (0.72–23.09)	0.11	-	-
EGFR	0.99 (0.51–1.90)	0.97	-	-
pAKT	0.95 (0.49–1.84)	0.88	-	-
p-ERK	0.90 (0.45–1.81)	0.77	-	-
pTEN	0.53 (0.24–1.17)	0.11	-	-
PIK3CA	4.18 (0.90–19.39)	0.07	-	-

OR: Odds Ratio; CI: Confidence Interval

## Discussion

Our study demonstrates that PIK3CA gene mutations and PI3K/AKT pathway activation are common events in TNBC, indicating a critical role of this pathway in TNBC pathogenesis.

Several population-based studies have analyzed the association between PIK3CA mutations and different molecular subtypes of BC with variable results. PIK3CA abnormalities have commonly been related to hormone receptors and HER2 overexpression, and PIK3CA mutations are known to be highly associated with Luminal-A phenotypes [15,35,37,38]. Nevertheless, recent studies on a wide series of BC have shown that PIK3CA mutations are recognizable in TNBC, even with low frequencies [21,22,39]. Recently, Kriegsmann et al. demonstrated a higher frequency of PI3K pathway alterations, comprising mainly PIK3CA mutations (22.1%), in a large series of TNBC [23].

Our results support Kriegsmann et al. experience, demonstrating that PIK3CA mutations are recognizable in a higher percentage (23.7%) of TNBC than previously reported in the literature [17,18,20–22]. Although histologic subtypes other than invasive ductal carcinoma are scarcely represented in our study, we could identify PIK3CA mutations in triple negative invasive lobular carcinoma, medullary carcinoma, and even in special variants of BC, as adenoid cystic carcinoma. These results underline the contribution of PIK3CA mutations in the biology of several histotypes of TNBC, confirming results of other authors [38,40].

To confirm the activating role of these mutations, we performed immunohistochemical analysis for PIK3CA downstream signaling pathways, such as pAKT and p-p44/42 MAPK, showing overexpression of these proteins in 100% of PIK3CA mutated TNBC. Interestingly, a strong association was identified between the presence of PIK3CA mutation and a higher score of pAKT (p = 0.0001), demonstrating the activating role of this genetic alteration in TNBC.

To perform a consistent evaluation of PI3K/AKT and BRAF/RAS/MAPK pathways, we analyzed other members of these pathways. In our experience, total loss of PTEN protein was detected in 11.3% of TNBC, all of these with basal-like phenotypes. pAKT and/or p-p44/42 MAPK were expressed in all TNBC with PTEN loss, according with the activating function of protein loss on these signaling pathways. Furthermore, PTEN loss was observed in association with both PIK3CA-mutated and PIK3CA-wild-type tumors.

A very recent study by Millis et al. on a wide series of TNBC described PTEN loss in 66% of tumors, which could be explained by different immunohistochemical thresholds related to staining intensity and percentages of cells (0+ or ≤50%) [22].

Although PTEN loss and PIK3CA mutations have often been reported as mutually exclusive [37], it should be noted that in more recent studies these genetic events are described as concordant in BC patients [18]. We hypothesize that combined mutations might be the consequence of sequential waves of mutations during clonal cell expansion, but it is also possible that PTEN loss and PIK3CA mutation might be responsible for different pathologic events in tumor progression.

In our experience, no mutations in EGFR exon 18–21, AKT1 exon 2, BRAF exon 15 and KRAS exon 2–3 were detected, in keeping with data from the literature [41–46].

The correlation analysis between PIK3CA status and TNBC molecular subtypes showed a statistically significant association between PIK3CA mutations and Luminal Androgen Receptor (LAR) subtype. Furthermore, the highest percentage of PIK3CA mutations was obtained in TNBC basal-like variants, in accordance with a very recent study that confirmed the significant relation between PIK3CA mutations and the presence of basal markers in a series of 75 TNBC [24]. However, no significant association was appreciable between PIK3CA mutations and TNBC basal-like or non-basal-like subtypes (p = 0.258). Recently, the Cancer Genome Atlas Network has shown that PIK3CA mutations were common in Luminal and HER2-enriched tumors, whereas they were the 2^nd^ most common mutation in basal-like BC. Moreover, activation of PI3K/AKT pathway was the highest in this subgroup [8].

Taken together, our results demonstrate that at least 33% of TNBC have deregulated PI3K/AKT pathways, making these pathways an attractive target for pharmacologic treatment and highlighting the importance of mutation profiling for individualized therapies. Indeed, there is a strong interest in developing rapid, reliable and sensitive methods which might be used for clinical routine detection of PIK3CA mutations in BC. In this study breast cancer samples (FFPE) were tested by Real-Time PCR. This approach is more sensitive than Sanger sequencing, having the ability to detect mutations at a level of greater than 5%, which are present only in a subgroup of cancer cells or in tumors with significant contamination. The higher frequencies of PIK3CA mutations detected in our and Kriegsmann et al. studies are related to more sensitive methodologies, widening the opportunities for TNBC patients to be appropriately selected for individualized targeted therapy strategies. Moreover, the primers used in the cobas® PIK3CA Mutation Kit were designed to avoid false positive results due to presence of PIK3CA pseudogene sequence mismatch at the end of exon 9. [47]. We applied cobas® PIK3CA Mutation Kit also for the analysis of a small series of non-TNBC tumors, showing 33.3% of mutations rate, namely 40% in Luminal-A-like subtypes, 25% in Luminal-B-like subtypes, and 33.3% in Her2 positive subtypes. Although the small number of tumors could not allow to reach any statistical significance, the results were similar to those in the literature, even in this small sized sample [8].

Conflicting results about the prognostic meaning of PIK3CA mutations are evident from the literature. Some authors reported better overall survival (OS) and disease free survival (DFS) in patients harboring these genetic alterations, compared to patients without mutations [18,35,36,38,48,49]. In contrast, other authors reported that PIK3CA mutations in BC patients were associated with poor clinical outcome [50–52].

Our data on prognostic significance of PIK3CA mutations, restricted to 65 patients due to incomplete 5-years OS information, could not demonstrate any significant association between PIK3CA mutations and OS. The association between clinical-pathological data and PIK3CA mutations on all the TNBC showed that higher grades and higher proliferation indexes were significantly associated with wild-type PIK3CA tumors.

Several drugs targeting multiple levels of the PI3K network, such as PI3K, AKT, and mTOR, have been progressively taken into consideration in clinical trials for BC [53]. However, the use of PI3K/AKT pathway inhibitors as single-agent therapies has been demonstrated minimally effective in some diseases, according to the complexity of the PI3K/AKT pathway, with activation of multiple feedback and cross-talk mechanisms that might explain this drug resistance. For this reason, PI3K pathway inhibitors are being tested in human trials in combination with HER2, MEK and ER inhibitors, supposing that the simultaneous targeting of these escape mechanisms will lead to clinically success [54–57].

Recent results of Janku et al. have shown the clinical significance of highlighting PIK3CA molecular status in BC patients, since tumors with PIK3CA mutations treated with PI3K/AKT/mTOR inhibitors showed a response rate of 30% matched to 10% in wild-type PIK3CA tumors; the response rate in wild-type PIK3CA tumors was comparable to previous reports (4%-11%) when patients were treated on phase I trials without molecular selection [58]. Moreover, Fink et al. found that gene expression profiles for TNBC subtype definition were poor predictors of response to kinase inhibitors, whereas high sensitivity was reported when treatment options were based on specific genetic abnormalities of tumor, such as PTEN loss or PIK3CA mutations [59].

In conclusion, our study contributes to defining the complexity of TNBC category and, considering the high percentage of genetic alterations involving PI3K/AKT pathways identified in our series, supports the necessity to subclassify TNBC on the basis of their specific genomic abnormalities, in order to appropriately select patients who would more likely benefit from current targeted therapeutic strategies. Moreover, our results highlight the issue concerning the application of reliable and sensitive methodologies to detect PIK3CA mutations in routine practice.
